# Precession dynamics and morphology of rolling samaras

**DOI:** 10.1098/rsif.2025.0391

**Published:** 2025-09-24

**Authors:** Breanna Marie Schaeffer, Andrew Keith Dickerson

**Affiliations:** ^1^ University of Tennessee Knoxville College of Engineering, Knoxville, TN, USA

**Keywords:** autorotation, tree seeds, dispersal

## Abstract

Samaras of species such as *Fraxinus americana*, *Fraxinus excelsior* and *Liriodendron tulipifera* exhibit a unique dual-axis descent characterized by spanwise rolling superimposed on vertical helical precession. While prior work has established the aerodynamic mechanisms of non-rolling samaras, *Acer* spp. serving as the classic example,, the aerodynamic role of rolling motion remains largely uncharacterized. Here, we present a comparative kinematic and morphological analysis of 30 rolling samaras across three species using high-speed multi-camera imaging and digital tracking. Our results reveal that all species maintain stable autorotation by rolling approximately seven cycles for each precession cycle, inducing periodic modulation of angle of attack and sinusoidal lift generation without observable wingtip wobble. Species-specific variation in wingspan, mass and thickness generates distinct descent velocities, but across all groups wing thickness emerges as the single most predictive morphological trait. We demonstrate that descent velocity scales inversely with thickness, with additional inverse power-law relationships linking thickness to both rolling and precessional angular velocities. Analytical modelling of lift-induced torque predicts negligible vertical oscillation, confirming observations. Samaras released into a vertical wind tunnel from a static position show that the time to stable autorotation strongly depends on the initial release orientation. In our observations, rolling begins before precession, and the stochastic direction of rolling sets the precessional direction.

## 1. Introduction

**Figure 1.**
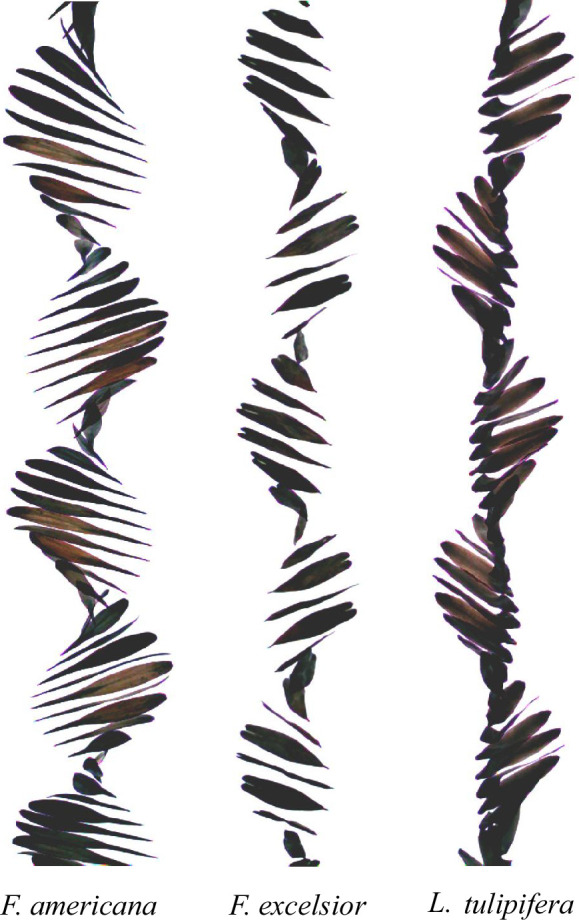
Composite image showing the descent of three different samara species—*Fraxinus americana*, *Fraxinus excelsior* and *L. tulipifera* captured at 1000 fps. The images illustrate the differences in falling trajectories and rotational motion among species.

Aerodynamic adaptations for improved seed dispersal are exemplified by samaras, winged seeds that exploit autorotation to enhance wind-assisted travel by prolonging time in flight [1,2]. Maple (*Acer* spp.) samaras descend solely through stable helical autorotation, whereas samaras from species such as *Fraxinus* spp. and tulip poplar (*Liriodendron tulipifera*) combine spanwise rolling with precession to produce a dual-axis rotation during descent [3] as seen in figure 1. Both rolling and non-rolling samaras follow helical trajectories but differ in the aerodynamic mechanisms that produce lift. While descent dynamics of maple samaras have been well characterized, the aerodynamic role of rolling motion remains largely unexplored, despite early observations recognizing that ash and tulip samaras rotate about both their spanwise and vertical axes [3].

Prior studies of autorotating samaras [4], fluttering plates [5,6] and tumbling parallelograms [7,8] reveal that morphological asymmetries and rotational dynamics significantly influence aerial trajectories. Fluttering involves oscillatory see-saw-like motion in which the falling plate does not flip, as is typical of falling paper [5,6]. Tumbling, in contrast, is characterized by end-over-end rotation driven by dynamic instabilities in aerodynamic torque [7,8]. Tumbling generates lift and lateral propulsion. Rolling is tumbling, but in the context of samaras, tumbling through a precessional rotation. The interplay between mass distribution, geometry and rotational kinematics governs which descent mode—fluttering, tumbling or rolling—is observed. Rolling samaras share descent characteristics with both non-rolling autorotators and tumbling plates.

Rolling motion introduces continuous reorientation of the wing relative to the airflow, fundamentally altering the aerodynamic forces compared to non-rolling autorotators. Unlike maple samaras, which maintain a relatively fixed angle of attack and generate lift through stable leading-edge vortices (LEVs) [9,10], rolling samaras experience periodic modulation of angle of attack as they rotate spanwise [11]. Dynamic rolling generates time varying lift force and torque, leveraging unsteady aerodynamic mechanisms similar to those observed in flapping insect flight [12].

The present study characterizes falling samara kinematics and links morphological features to descent dynamics. Using high-speed tracking of multiple species, we characterize three-dimensional (3D) trajectories, rolling and precession velocities and angle of attack modulation during descent. Mass, area and thickness are analysed to identify how morphological variation shapes flight performance. By quantifying rolling kinematics and uncovering the critical role of wing thickness in regulating descent velocity, the study addresses gaps in understanding the aerodynamic principles underlying rolling samara dispersal.

## 2. Methods

**Figure 2.**
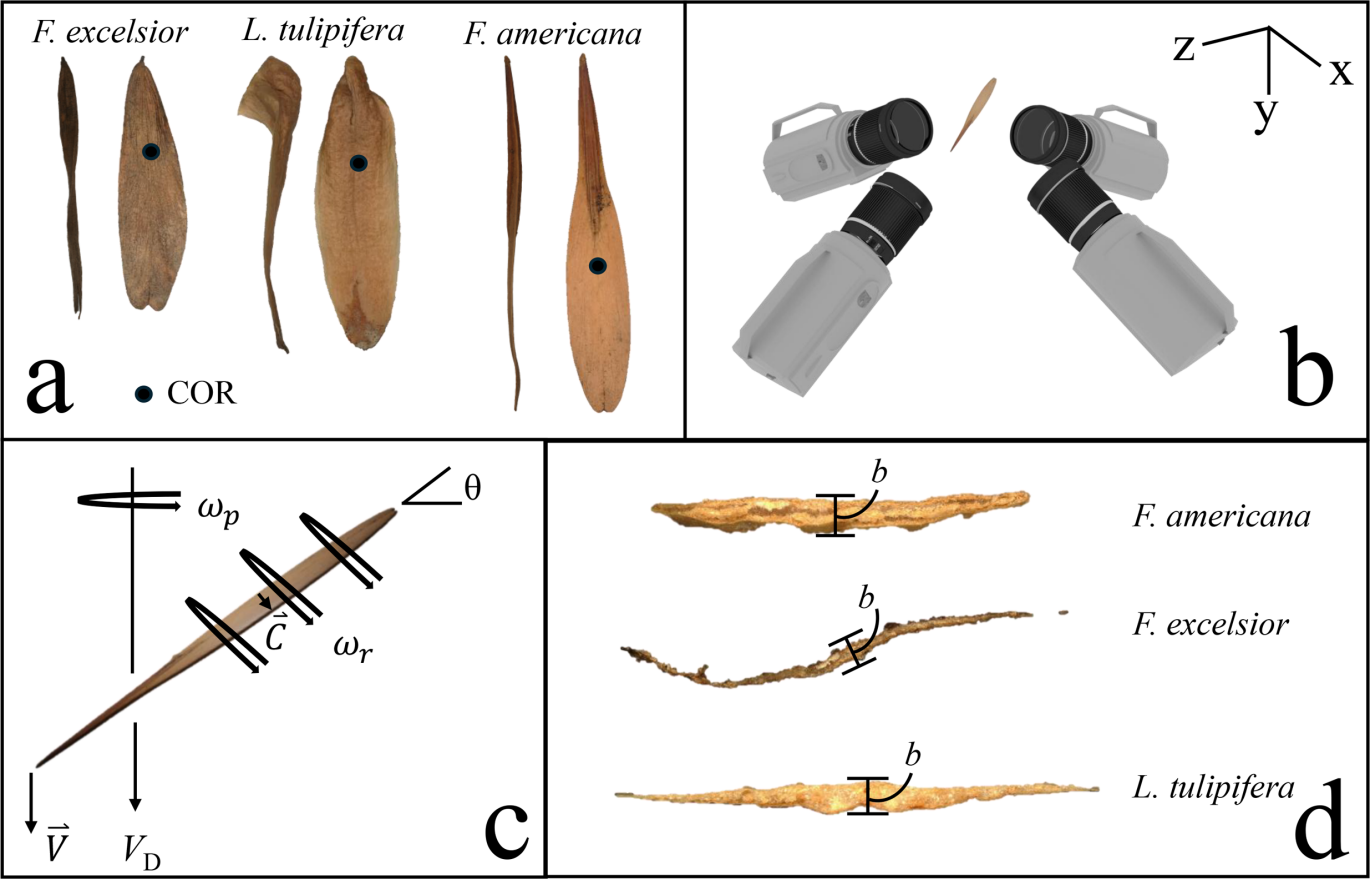
Overview of the samara species and experimental set-up. (a) Representative samaras from the three focal species: *F. americana*, *F. excelsior* and *L. tulipifera*. Black dots represent the approximate COR. (b) Experimental set-up for high-speed video tracking of falling samaras using four synchronized cameras. (c) Kinematic and dynamic flight variables analysed in this study, including rotational velocity ω, coning angle θ, rolling velocity ϕ and descent velocity $V_{\text{d}}$. (d) Cross-sectional views of samara seeds illustrating variations in thickness b.

Samaras from three species—*Fraxinus* (*F*.) *americana* L., *F. excelsior* L., and *L*. *tulipifera* L.—are collected in Knoxville, TN, after natural dispersal. Only visually undamaged samaras are selected for measurement and experimentation. Representative examples of each test species are pictured in figure 2a.

Samara mass m is measured using a Sartorius Secura 225D-1S analytical balance with a resolution of 0.0001 g before flight trials. Samara area A, span S and chord c are measured from still images as described in Schaeffer *et al.* [13]. Thickness b is measured at the point of maximum chord using a Mitutoyo caliper with 0.01 mm resolution.

Kinematic tracking of falling samaras is conducted using four high-speed video cameras, as illustrated in figure 2b. The analysis of the video recordings was performed using [14] DLTdv. The extracted 3D position data are used to compute kinematic parameters, including rotational velocity $\omega_{\text{p}}$, coning angle θ, rolling velocity $\omega_{\text{r}}$ and descent velocity $V_{\text{d}}$, using MATLAB. The measured values are summarized in figure 2c.

To assess the impact of initial orientation on flight dynamics, samaras are held manually at specified orientations before being released into a wind tunnel, as described in Schaeffer *et al.* [15]. Airflow is adjusted to match stable levitation velocity, allowing for a controlled comparison of automation initiation times and trajectories.

## 3. Results and discussion

We examine the morphology and descent kinematics of 30 rolling samaras, 10 each from three species: *F. americana*, *F. excelsior* and *L. tulipifera*, with three replicate flights of each specimen. The samaras of this study roll about their span axis as they helically precess downward. Such a precession is similar to that of non-rolling samaras [13]. When released, samaras begin to roll before the onset of precession. The stochastic roll direction sets the direction of precession, clockwise or counterclockwise. Our tulip and ash samaras always roll such that the bottom chordal edge rolls forward, similar to a fluttering, translating plate [8], as schematized in figure 2c.

### 3.1. Samaras roll without wobble

**Figure 3.**
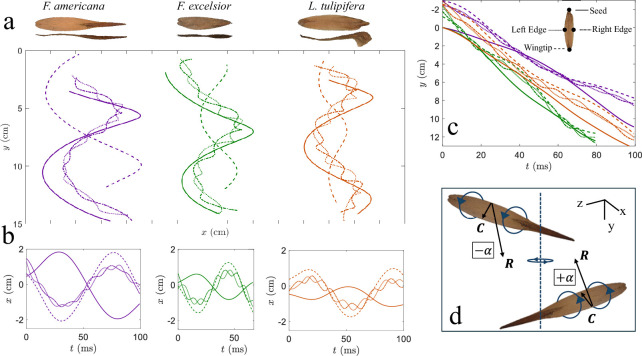
Kinematic and dynamic behaviour of rolling samaras. (a) x–y-projections of helical descents, tracking the seed, wingtip and chordal edges. (b) Temporal x-projections of descent show graphical comparisons of COR and rolls per precession cycle. (c) Temporal y-projections of descent show smooth tracks of seed and wingtip. (d) Representative orientations of a rolling samara showing positive +α and negative −α.

To characterize descent behaviour, we track the position of four key points on rolling samaras: the seed tip, wingtip, left chordal edge and right chordal edge. The 3D trajectories of representative samaras from each species are shown in figure 3a. Once initiated, the rolling direction dictates the direction of precession. The roll propels the samara forward [8], but the seed is more massive and generates less propulsive force than the wing. The result is a circular rotation rather than the linear translation exhibited by symmetric plates. When the seed is removed, rolling and precession still occur, but the radius of precession increases, indicating that the centre of aerodynamic rotation shifts further outward in the absence of the mass asymmetry. Findings from maple seed analogue models similarly show that minor shifts in mass distribution can dramatically alter the descent trajectory from circular to compound paths [16].

Despite qualitatively similar descent dynamics across our three test species, morphological variation is stark. The heaviest species we test have the smallest wing area. Plan and side view photos are shown in figure 2a, while mass and area measurements are tabulated in table 1. Broad morphological variation drives significant differences in descent velocity $V_{\text{d}}$, precession angular velocity $\omega_{\text{p}}$ and rolling angular velocity $\omega_{\text{r}}$, tabulated in table 1. Rolling and precession angular velocities are tightly coupled, however, with an average across all trials of 5.2±1.1 rad s^−1^, number of trials N=90.

**Table 1.** Summary of rolling samara kinematics and morphology. Each species includes N=10 individuals, for a total of 30 samples. The final row reports mean values across all species.

species	*b* (mm)	*m* (mg)	*A* (cm²)	*V*_d_ (cm s^−1^)	$\omega_{r}$ (rad s^−1^)	$\omega_{\text{p}}$ (rad s^−1^)
*F. americana*, N= 10	0.50±0.02	39.0±3.0	1.867±0.142	131±9	442.6±59.7	79.6±6.8
*F. excelsior*, N= 10	0.41±0.02	60.0±10.0	1.784±0.115	191±21	617.6±76.6	119.3±13.4
*L. tulipifera*, N= 10	0.46±0.02	47.0±5.0	2.141±0.183	148±15	503.5±66.4	93.5±16.6
mean	0.46±0.03	50.4±8.5	1.88±0.21	158±23	521±96	97±18

Tabular values can be compared graphically by temporal representations of position. We plot lateral displacement of the tracked points in x versus time as shown in figure 3b. The amplitude and frequency of oscillations in x are influenced by the span of the samara, with the relative rapidity in the rotation of *F. excelsior* being cursorily explained by its stubbiness compared to the other two species. Lateral displacement of the seed tip indicates a centre-of-rotation (COR) located closer to the wingtip. We estimate COR locations from videos and provide labels in figure 2a. Among the studied species, the COR in *F. americana* is located nearer mid-span, while *F. excelsior* and *L. tulipifera* have a COR is closer to the seed.

We plot the temporal vertical displacement of the seed, wingtip and chordal edges for three representative cases in figure 3c. The seed follows a relatively smooth vertical trajectory, as its higher mass concentration attenuates local oscillations, maintaining a smooth helical path [17,18]. A subtle shift in the coning angle, obscured in the full descent profile shown in figure 3a, becomes apparent when examining the vertical trajectories of each tracked point in figure 3c. Similar behaviour has been reported in other autorotating systems, where uneven mass distribution leads to periodic aerodynamic adjustments and variability in descent trajectory [19]. Across all test species, the average descent velocity $V_{\text{d}}$ is 153±32 cm s^−1^, N=90. By comparison, maple samaras (*Acer* spp.), which do not exhibit rolling, descend more slowly at 102±9 cm s^−1^ [13,20].

Prior studies of autorotating seeds and flapping insects have shown that unsteady aerodynamic features—such as vortex shedding and lift oscillations—can promote flight stability rather than sensitivity [11,12]. Rolling samaras appear to leverage similar dynamics, using periodic changes in angle of attack to maintain controlled descent. We assert that the magnitude and timescale of lift oscillations are too small to interfere with precession, which is explored below, and therefore the cause for the aforementioned coning angle oscillations, with a frequency that does not match $\omega_{\text{r}}$, is unknown but may be the result of transients incurred during samara release.

Unlike flapping wings [21,22], which oscillate and periodically reverse their angle of attack α, rolling samaras undergo continuous spanwise rotation that steadily modulates α. The resulting periodic fluctuations reflect continuous rolling, rather than true reversals in wing–airflow alignment. Angle of attack α is periodic and discontinuous, transitioning from $90^{\circ }$ to $-90^{\circ }$ upon the wing surface normal aligning with the airflow vector. The angle of attack is generically defined as


(3.1) $$\begin{array}{lcr} & \alpha =\tan^{-1}\left(\frac{\left\|R\|\|C\right\|}{R\cdot C}\right), & \end{array}$$

where $R=\tan^{-1}(V_{d}/0.75S\omega_{p})$ is the local velocity vector and C is the vector between the chordal edges. Representative orientations of a rolling samara with positive (+α) and negative (−α) angles of attack, defined relative to the local direction of motion R, are shown in figure 3d. In contrast to rolling samaras, maple samaras (*Acer* spp.) maintain nearly constant angles of attack between $20^{\circ }$ and $40^{\circ }$ during descent [4,13,23]. Insects such as bumblebees [24], dragonflies [25] and beetles [26] maintain high angles of attack between $30^{\circ }$ and $60^{\circ }$, enabling unsteady lift mechanisms such as delayed stall and LEVs [27]. Rolling samaras may leverage analogous aerodynamic effects through passive, cyclic modulation of angle of attack. Periodic fluctuations in α can regulate descent by adjusting lift across each rotation, a mechanism also observed in biological flyers such as mosquitoes [28].

Rolling induces small-amplitude perturbations superimposed on the dominant precession, introducing unsteady aerodynamic effects that should, intuitively, result in vertical oscillation of the wingtip [7]. However, we observe no such wobble of the wingtip. Prior studies of autorotating and fluttering plates suggest that the lift coefficient $C_{L}$ during periodic motion can be approximated as a sinusoidal function [7]. Following Fang *et al.* [11], we can write the lift force due to rolling as


(3.2) $$\begin{array}{lcr} & F_{L,r}(t)=\frac{1}{2}\underset{C_{L}}{\underbrace{\left(C_{1}\sin\left(\frac{2\pi t}{T}\right)+C_{2}\right)}}\rho V_{\text{d}}^{2}A, & \end{array}$$

where *ρ* = 1.23 kg m^−3^ is air density, and constants [11] $C_{1}=0.9$ and $C_{2}=0.7$. Equation (3.2) mandates we assume $d\omega_{\text{r}}/dt=0$, which is confirmed by kinematic tracks (figure 3b). Assuming this force acts at a moment arm of S/4 from the wingtip, the corresponding torque is


(3.3) $$\begin{array}{lcr} & \tau_{\text{max}}=\frac{S}{4}F_{\text{max}}. & \end{array}$$

The moment of inertia for a thin plate of uniform density about its short axis is $mb^{2}/12$. We assume that the samara will pivot S/4 from the seed tip due to fluctuation in lift. The moment of inertia relevant to roll-induced lift can be estimated by the parallel axis theorem:


(3.4) $$\begin{array}{lcr} & I=\frac{1}{12}mb^{2}+m{\left(\frac{S}{4}\right)}^{2}. & \end{array}$$

The maximum angular acceleration induced by lift fluctuation is then


(3.5) $$\begin{array}{lcr} & {\dot{\omega }}_{\text{max}}=\frac{\tau_{\text{max}}}{I}=\frac{3(C_{1}+C_{2})\rho V_{\text{d}}^{2}AS}{2mb^{2}+6mS^{2}}, & \end{array}$$

where ${\dot{\omega }}_{\text{max}}\approx 197\;{\text{rad s}}^{-2}$, using representative averages from *L. tulipifera*: *V*_*d*_ = 131 cm s^−1^, projected area $A=2.141{\text{cm}}^{2}$, span S=3.9cm, mass m=47mg and wing thickness b=0.46mm.

To estimate the vertical displacement generated by ${\dot{\omega }}_{\text{max}}$, we assume the acceleration acts over a quarter roll cycle. The resulting vertical displacement of the wingtip is


(3.6) $$\begin{array}{lcr} & y_{\text{max}}=\frac{3}{32}S\pi^{2}\frac{{\dot{\omega }}_{\text{max}}}{\omega_{\text{r}}^{2}}, & \end{array}$$

which yields 27 µm for $\omega_{\text{r}}=510.2$ rad s^−1^. Such a minuscule displacement at the wingtip, an estimate that neglects added mass effects, explains why the roll does not visibly wobble the samara wingtip throughout precession.

### 3.2. Wing thickness is the prevailing dimension governing descent velocity

Our three focal species—*F. americana*, *F. excelsior* and *L. tulipifera*—illustrate that rolling-autorotation can occur across a range of samara shapes. Tulip poplar (*L. tulipifera*) samaras are dry, woody, winged fruits with a span of 3.6±0.3 cm, N=10, each derived from a single carpel within an upright conical aggregate that disaggregates at maturity in late autumn [29,30]. *Liriodendron tulipifera* samaras have bulbous seeds and strongly curved wings extending dorsally from the upper edge. Each unit in the conical aggregate contains one or two samaras that feature a dorsally extended wing that facilitates wind dispersal over distances up to five times the height of the parent tree [29–31]. In contrast, ash samaras exhibit flat, teardrop-shaped seeds [32]. The planar wings of *F. americana* taper symmetrically, while *F. excelsior* samaras have a subtly helical wing, introducing spanwise curvature (figure 2a). Morphological differences among our two ash species align with previous studies highlighting substantial interspecific and intraspecific variation in wing length, area and seed-to-wing proportions [3,11,33]. Despite variability in form, all three species maintain an aspect ratio S/c averaging 4.5±0.7 and seed-biased mass distribution that promotes autorotation. Despite their morphological differences, our three focal species share a surprising aerodynamic commonality; thickness b emerges as the most predictive trait shaping flight performance.

One might expect conventional length-based scaling laws to predict differences in aerodynamic behaviour because many aerodynamic traits scale predictably with a characteristic length *L* [34,35]. In allometric scaling, planform area scales as $A∼L^{2}$, and mass scales volumetrically as $m∼L^{3}$. Such assumptions are widely applied to engineered rotors [36–38] and biological structures, including seed wings [13], to estimate drag, lift and dispersal potential [4,39]. Across all three species in this study, however, neither the square of span $S^{2}$ nor chord length $c^{2}$ strongly correlates with area (*R*${,}^{2}<0.1$; electronic supplementary material, figure S1), challenging the intuition that planform dimensions reliably predict size and weight.

**Figure 4.**
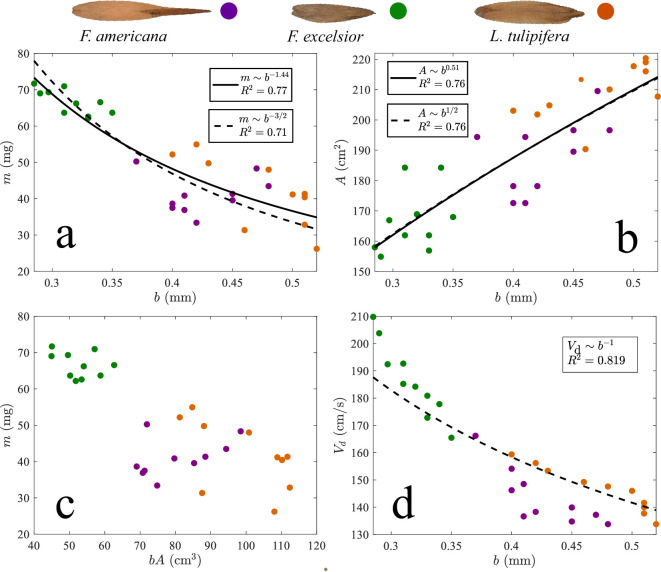
Scaling relationships in rolling samaras. (a) Mass m versus wing thickness b. (b) Planform area A versus b. (c) m versus bA. (d) Descent velocity $V_{\text{d}}$ versus b.

Instead, both mass and planform area exhibit strong scaling relationships with thickness for all test species. Mass follows an inverse power-law scaling based on best-fit regressions, with $m∼b^{-1.44}$ ($R^{2}=0.811$), as shown in figure 4a. Planform area scales positively with thickness, following $A∼b^{0.46}$ ($R^{2}=0.752$), as seen in figure 4b. In short, the samaras with thinner wings are the heaviest. Although the relationships shown in figure 4 appear approximately linear on a log–log scale, the regressions are best fitted by power-law models, which yield higher $R^{2}$ values than simple linear regressions.

The unexpected relationships plotted in figure 4a,b suggest that structural integrity is not the primary factor driving thickness. On the contrary, a plot of bA versus m shows that rolling samaras are volume-conserving, with each species having a slightly different density, as shown in figure 4c. Rather than distributing material uniformly, samaras concentrate structural reinforcement in the reproductive seed and central spine, while maintaining thinness at wing edges (figure 2d). Strategic allocation of material in these regions likely enhances stability and mechanical efficiency without compromising aerodynamic function [4,39,40], but such an investigation is a topic for future simulation. Comparable design principles appear in engineered flight systems, where internal spars or composite reinforcements provide stiffness without increasing thickness throughout [41–43].

In light of the unexpected morphological scaling with thickness, we next consider the role of thickness and wing area on descent velocity $V_{\text{d}}$. In a previous study [13], we successfully united the descent velocity of nine *Acer* samara species using a classical drag model that combines form drag and lift:


(3.7) $$\begin{array}{lcr} & mg∼\frac{1}{2}C_{D}\rho V_{\text{d}}^{2}A\;\cos\;\theta , & \end{array}$$

where g is gravitational acceleration, and $C_{D}$ is the drag coefficient. Our above best-fit scalings, $m∼b^{-1.44}$ and $A∼b^{0.46}$, are very close to more convenient choices for scaling exponents. We thus choose $m∼b^{-3/2}$ ($R^{2}=0.77$) and $A∼b^{1/2}$ ($R^{2}=0.73$) for substitution into equation (3.7). The result provides


(3.8) $$\begin{array}{lcr} & V_{\text{d}}∼\frac{1}{b}, & \end{array}$$

plotted in figure 4d, with $R^{2}=0.85$. The emergence of a simple, predictive relationship between thickness and descent velocity reveals that thickness plays a fundamental and previously underappreciated role in rolling samara flight. The establishment of equation (3.7) challenges the traditional view of relating terminal velocity to wing loading $V_{\text{d}}∼(mg/A{)}^{1/2}$, most notably proposed by Ausperger [44]. We defend the use of equation (3.7) by plotting $V_{\text{d}}$ versus $(mg/A{)}^{1/2}$ for our data alongside the rolling samara data from Ausperger [44], Greene & Johnson [45] and Azuma & Yasuda [46] in electronic supplementary material, figure S2.

**Figure 5.**
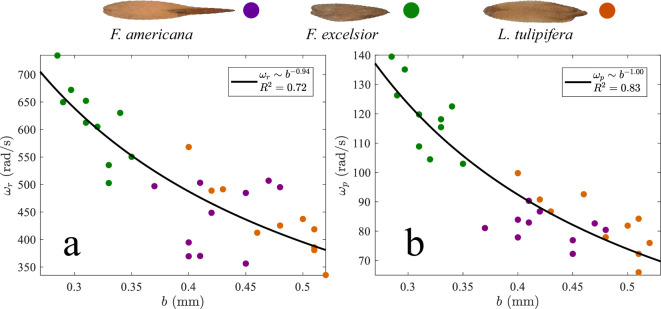
Relationship between wing thickness b and (a) rolling velocity $\omega_{\text{r}}$ and (b) precession velocity $\omega_{\text{p}}$.

The aerodynamic importance of thickness parallels findings in tumbling plate dynamics [8,47], where thickness influences rotational stability and descent behaviour. Experimental [5] and computational [8] studies of falling plates have shown that increased wing thickness suppresses chaotic or erratic motion and instead promotes periodic, stable rotation. Observations of rolling samaras reveal a similar trend. Rolling velocity decreases with thickness ($\omega_{\text{r}}∼b^{-0.84},\;R^{2}=0.72,\;N=30$), an observation that is species independent, as shown in figure 5a. precession velocity also decreases with thickness ($\omega_{\text{p}}∼b^{-0.90},\;R^{2}=0.83,\;N=30$), as shown in figure 5b. If we approximate the best fit in figure 5b as $\omega_{\text{p}}∼b^{-1}$, equation (3.8) provides $V_{\text{d}}∼\omega_{\text{p}}$, an intuitive result. Seed descent traits such as morphology and terminal velocity influence not only flight dynamics but also ecological outcomes. Interspecific variation in descent rate has been shown to influence mean dispersal distance, with consequences for seedling establishment and overall plant fitness [44], suggesting that the aerodynamic differences reported here may carry important ecological implications.

### 3.3. Release position catalyses rolling proceeding precession

Rolling, and thus precession, is more rapidly initiated from some static release positions than others. We filmed the release of three specimens from each species, with three replicates at each of five distinct starting orientations. Releases are initiated from the top of a vertical wind tunnel (§2). The wind tunnel provides a terminal velocity field that hastens the onset of stable autorotation in view of our high-speed camera. For each of 135 trials, we measure two key timepoints. The first is the time to complete one full rolling cycle, $t_{r}$. The second is the time to stable precession, $t_{p}$, defined as the time to levitation without further descent in the laboratory frame. We acknowledge that the method for determining $t_{r}$ and $t_{p}$ involves human interpretation during a frame-by-frame analysis, introducing a degree of subjective error, and estimate this error to be <40 ms, or half the precession period of our slowest rotator. The qualitative responses to each initial orientation are consistent across all three species, enabling the generalization of behavioural trends without species-specific distinctions. Electronic supplementary material, videos S1–S5 , are provided for each orientation to illustrate the observed responses.

**Figure 6.**
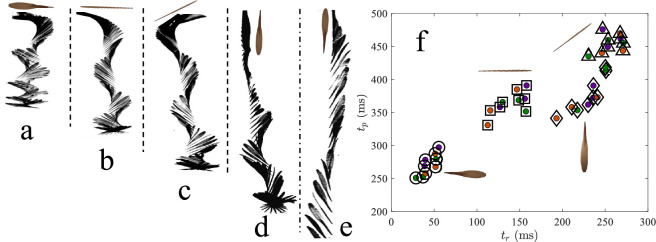
Initiation of autorotation in rolling samaras. (a–e) Sequential images illustrating different initial orientations of samaras relative to the airflow: (a) wing parallel to the flow, (b) wing perpendicular to the flow, (c) wing at a 45° angle to the flow, (d) wing parallel to flow with seed up and (e) wing parallel to airflow with wingtip up. (f) Relationship between the time to complete one full roll ($t_{r}$) and the time required to achieve stable precession ($t_{p}$). Release orientation influences the time to steady autorotation.

The first orientation positions the samara chord parallel to gravity and span parallel to the horizontal, minimizing aerodynamic resistance at release, as depicted in figure 6a (electronic supplementary material, video S1). In this configuration, the seed leads the descent, causing the wingtip to rotate upward. Rolling motion is initiated almost immediately, with an average time to roll of $t_{r}=47\pm 8\;\text{ms}$. Time to precession is also rapid, occurring within $t_{p}=262\pm 13\;\text{ms}$. All reported initiation times reported herein should be interpreted for the sake of comparison and are not representative of transients in natural conditions because the samaras experience a terminal velocity field immediately at release. Therefore, we expect natural entry into autorotation to be longer. Among all tested configurations, this orientation consistently produced the fastest transition into stable autorotation, confirming its aerodynamic favourability.

The second orientation positions the chord parallel with the horizontal, maximizing aerodynamic drag at release. In this case, rolling motion does not begin immediately; instead, the seed initially drops downward before rolling begins. Average time to roll increases to $t_{r}=120\pm 15\;\text{ms}$, while time to precession occurs at $t_{p}=330\pm 20\;\text{ms}$, as seen in figure 6b. Once rolling begins, the transition to stable precession proceeds rapidly. A similar sequence occurs in the third orientation, where the wing is tilted at a $45^{\circ }$ angle to the horizontal from the second orientation. The seed initially flips downward, causing a brief delay in rolling initiation that can be attributed to the time it takes for the seed to rotate down. Time to roll in this case increases further to $t_{r}=190\pm 18\;\text{ms}$, and precession is achieved at $t_{p}=390\pm 22\;\text{ms}$, as shown in figure 6c.

In the fourth orientation, the span is vertical with the seed facing upward. The samara flips seed down to delay rolling compared to other orientations. Average time to roll is $t_{r}=245\pm 14\;\text{ms}$, and stable precession develops after approximately $t_{p}=460\pm 18\;\text{ms}$, as seen in figure 6d. The fifth and final orientation positions the span vertically with the seed down. Intermittent rolling occurs throughout the descent, but the motion remains irregular. The samara does not fully transition to stable precession within our approximately 55 cm high tunnel viewing area, despite windspeed being set at the terminal velocity of the samara, as shown in figure 6e.

Samaras that initiate rolling earlier ($t_{r}$) reach a steady precession ($t_{p}$) more quickly, as shown in figure 6f. In natural conditions, samaras are unlikely to detach from the parent tree in the ideal orientation. Branch motion, wind disturbances and variability in fruit abscission mechanics likely result in a wide distribution of initial orientations at release [45]. However, it has been shown that symmetric samaras released into a crosswind initiate rotation more rapidly than those in still air [48], indicating that orientation may be less critical than our results indicate.

Field observations have shown that tulip and ash samaras often fall in non-ideal orientations and may briefly descend without rotating before aerodynamic forces reengage and stabilize the flight path [3]. In these species, seed release occurs over an extended dispersal season—often lasting several weeks to months—during which environmental conditions such as wind, humidity and temperature can vary substantially, increasing the likelihood of non-ideal release scenarios [45]. In contrast, species such as *Acer* exhibit more temporally synchronized dispersal, with samara release typically concentrated within a narrow window of just a few days to a week [49], thereby limiting exposure to such variability. The observed consistency in rolling initiation across all species, even from suboptimal starting configurations, suggests that rolling samaras possess an inherent aerodynamic robustness that enables recovery from a range of initial states. Aerodynamic recovery from unfavourable orientations enhances time aloft and dispersal distance. Selection may therefore favour geometries that not only optimize steady-state flight but also promote reliable initiation of autorotation. Orientation-dependent initiation dynamics appear to be a critical, yet unstudied, component of the passive dispersal performance of rolling samaras.

## 4. Conclusion

In this study, we investigate the descent dynamics of rolling samaras from three species— *F. americana*, *F. excelsior* and *L. tulipifera*—by detailing their kinematics and establishing scaling relationships that connect morphology to kinematics. Using high-speed imaging and quantitative tracking, we measured rolling velocity, precession velocity and time to autorotation in a terminal velocity field for various release orientations. The angle of attack of the rolling wing oscillates between −90° and 90° and is discontinuous. The resulting sinusoidal lift force occurs over a timescale too brief to wobble the samara throughout its rolling period. The result is a smooth helical path drawn vertically by both seed and wingtip.

Morphological and kinematic data reveal that wing thickness is the most predictive morphological feature influencing flight performance. Thicker wings correspond to greater mass and higher descent velocity, and both quantities scale consistently with thickness across species. Rotational velocity measurements further support the role of thickness in flight dynamics. Both rolling velocity and autorotational velocity decrease with thickness, indicating that thicker samaras rotate more slowly.

Upon release, more rapid initiation of rolling leads to a shorter period to establish precession, as the rolling motion reorients the wing relative to the incoming airflow. Samaras that are released with the seed down and span parallel to gravity fail to achieve autorotation in our tunnel. All other orientations produce autorotation in less than 500 ms. We posit that natural startup periods will be longer due to the absence of a terminal velocity field at release. Nevertheless, rolling samaras demonstrate a remarkable ability to enter autorotation from a vast range of release conditions.

## Data Availability

Data are available on Zenodo [50]. Electronic supplementary material is available online [51].
